# Supplementary material for: Guided anisotropic oxygen transport in vacancy ordered oxides
Source: Nat Commun. 2023 Sep 28;14:6068. doi: 10.1038/s41467-023-40746-4 (PMC10539514; doi:10.1038/s41467-023-40746-4)
Supplement: Supplementary file 1 — Supplementary Information [file 41467_2023_40746_MOESM1_ESM.pdf]

Supplementary Information for

Guided anisotropic oxygen transport in vacancy ordered oxides

Zhenzhong Yang[1,2†], Le Wang[1,†], Jeffrey A. Dhas[3,4], Mark H. Engelhard[3], Mark E. Bowden[3], Wen Liu[3,5], Zihua Zhu[3], Chongmin Wang[3], Scott A. Chambers[1], Peter V. Sushko[1,*], and Yingge Du[1,*]

[1]Physical and Computational Sciences Directorate, Pacific Northwest National Laboratory, Richland, WA 99354, USA

[2]Key Laboratory of Polar Materials and Devices (MOE), Department of Electronics, East China Normal University, Shanghai, 200241, P. R. China

[3]Environmental Molecular Sciences Laboratory, Pacific Northwest National Laboratory, Richland, WA 99354, USA

[4]School of Chemical, Biological and Environmental Engineering, Oregon State University, Corvallis, OR, 97331, USA

[5]State Key Laboratory of Biogeology and Environmental Geology, China University of Geosciences, Wuhan, 430074, China

*E-mail: peter.sushko@pnnl.gov; yingge.du@pnnl.gov

Supplementary Note 1

For BM-SFO on STO, the half-order peaks appear in the out-of-plane XRD θ-2θ scan (Supplementary Fig. 1), originating from the alternate stacking of FeO_6 octahedral and FeO_4 tetrahedral layers along the out-of-plane direction (OVCs parallel to the substrate surface), which is consistent with our STEM observation shown in Fig. 1e. By contrast, the absence of half-order peaks for BM-SFO on LSAT suggests that OVCs are vertical to the substrate surface, which is further confirmed by our STEM image shown in Fig. 1f.

Supplementary Note 2

Atomic structures (including O) of the pristine BM-SFO and new formed P-SFO can be clearly demonstrated by aberration-corrected HAADF and annular bright-field (ABF)-STEM. The oxygen vacancy ordering of BM-SFO is clearly visible in Supplementary Fig. 3a. Since the contrast of ABF images exhibits a $Z^{1/3}$ dependence (Z being the atomic number), they are more sensitive to lighter atoms, such as lattice oxygen. In Supplementary Fig. 3c, the line profile corresponding to ABF-STEM image (Supplementary Fig. 3b**)** shows alternating intensities for oxygen layers, revealing that each FeO_4-connected OVC is sandwiched by stoichiometric FeO_6 layers, consistent with the HAADF-STEM image. In contrast, for the P-SFO phase, such modulation disappears in the HAADF (Supplementary Fig. 3d) as well as the O intensity line profile (Supplementary Fig. 3f), indicating the O ions insertion into OVCs. As P-SFO is unstable under high temperature and sensitive to the electron beam[1-3], the slight octahedral distortions in Supplementary Fig. 3e are most likely due to the slight reduction of P-SFO. Theoretical studies also suggested that the existence of V_Os in $SrFeO_x$ may lead to such structural asymmetry[4]. A clear energy shift in electron energy loss spectra (EELS) (Supplementary Fig. 3g) is observed for BM-SFO and P-SFO, further confirming the oxidation of Fe^{3+} to Fe^{4+}.

Supplementary Figures

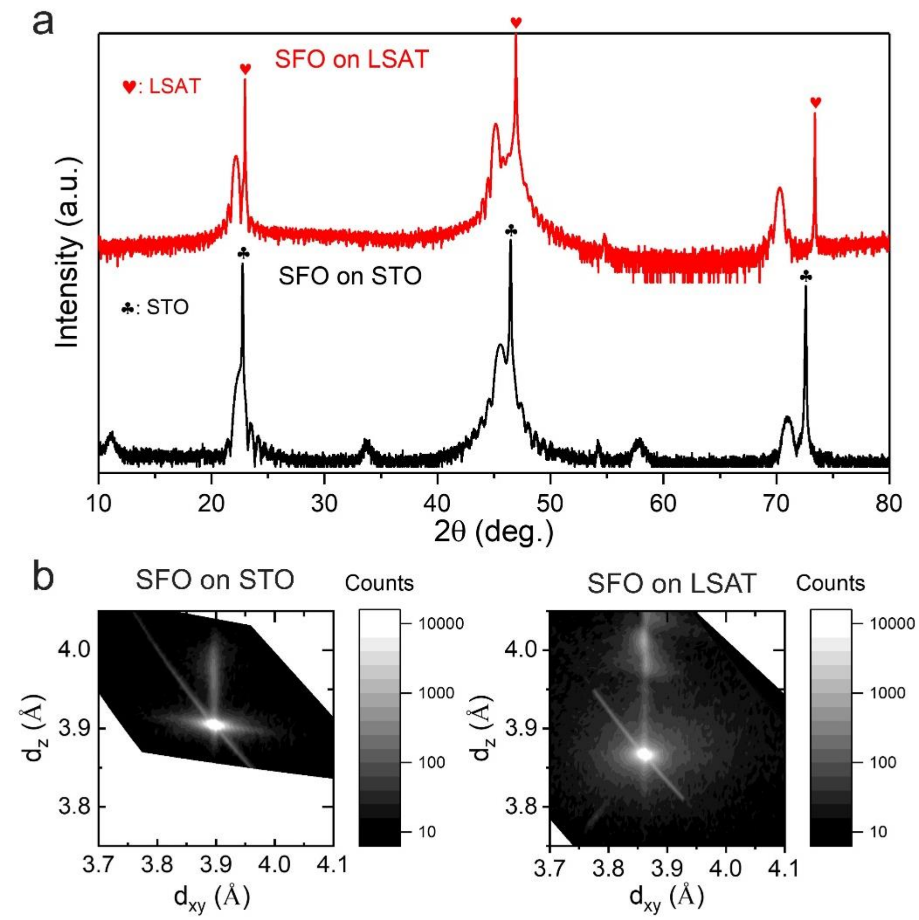

Supplementary Figure 1 | Structural characterization of epitaxial BM-SFO thin films. a, High-resolution XRD out-of-plane θ-2θ scans for BM-SFO grown on STO and LSAT. The STO and LSAT substrate peaks are marked with '♣' and '♥', respectively. **b**, Reciprocal space maps near the (103) reflection for these two samples, confirming structural coherence.

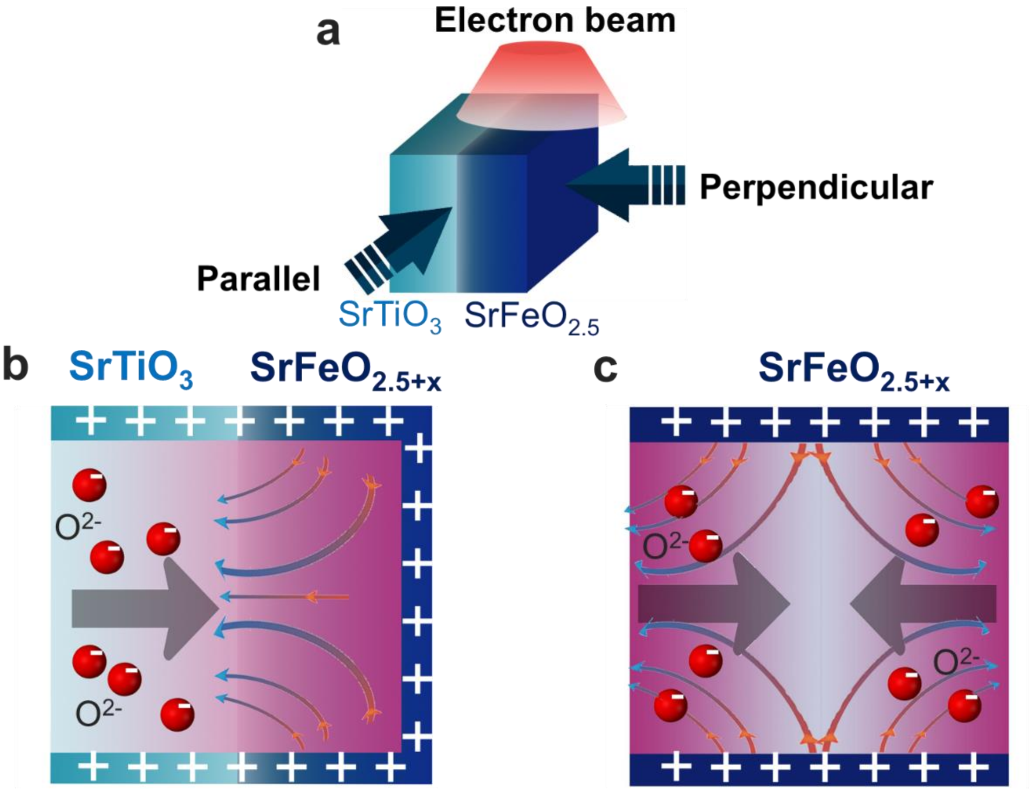

Supplementary Figure 2 | Effect of the electron beam on the O ions diffusion. a, Schematics of the sample orientation relative to the electron beam and viewing directions parallel (**b**) and perpendicular (**c**) to the interface. **b**, Schematics of electric field component perpendicular to the interface (shown in the viewing direction parallel to the interface) provides the driving force for O^{2-} ions diffusion from STO to SFO. **c**, Schematics of electric field component parallel to the interface (shown in the viewing direction perpendicular to the interface) provides the driving force for O^{2-} ions diffusion along the SFO film.

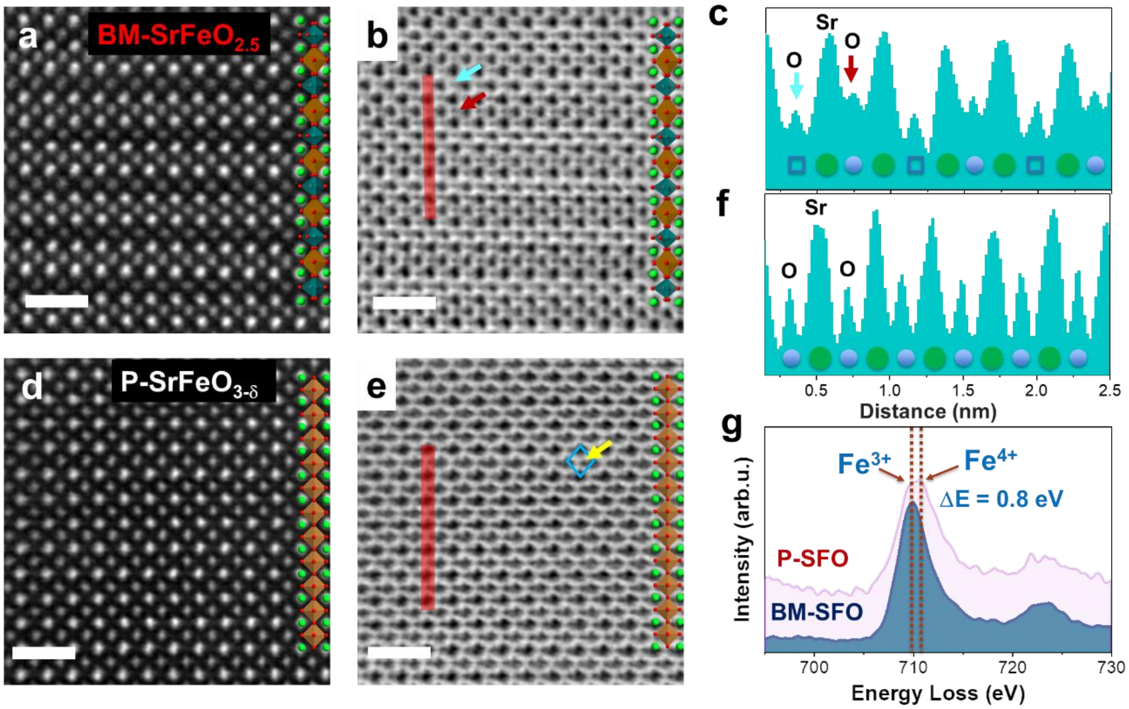

Supplementary Figure 3 | Evolution of the lattice structure and oxygen occupancy in BM-SFO and P-SFO phases. HAADF and ABF-STEM images for the pristine state BM-$SrFeO_{2.5}$ (**a**, **b**) and O rich P-$SrFeO_{3-\delta}$ (**d, e**). Line intensity profiles of inversed ABF contrast (**c** and **f**) corresponding to red lines in ABF images of **b** and **e**, respectively. The small hollow blue squares and purple spheres are used to indicate the half and fully occupied oxygen sites, respectively. The large green spheres indicate the Sr columns. The scale bars in **a**, **b**, **d**, and **e** are 1 nm. **g,** EELS of Fe $L_{2,3}$ edge shows the valence change during the phase transformation from BM-SFO to P-SFO.

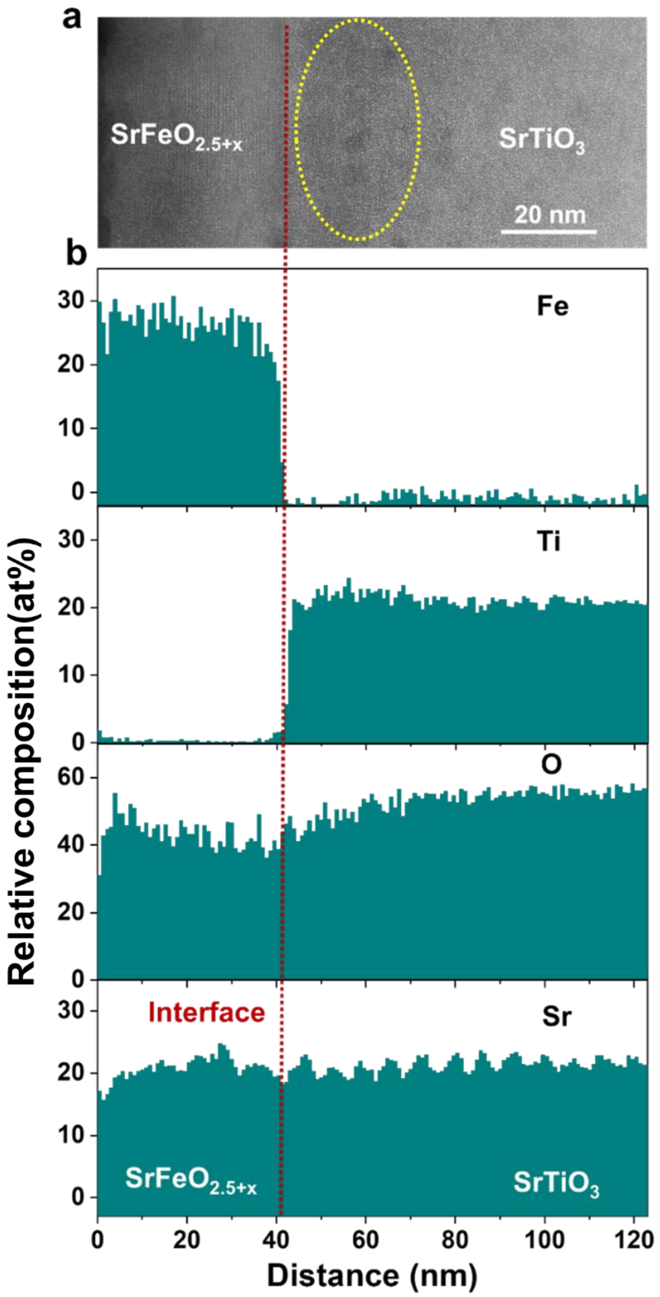

Supplementary Figure 4 | Elemental composition profiles across SFO/STO interface subjected to simultaneously applied heating and in situ TEM beam illumination. a, HAADF-STEM image of the SFO/STO sample after the in situ TEM experiment (simultaneously applied 300 °C sample heating and electron beam illumination) was completed. **b**, Relative composition of O, Fe, Ti, and Sr estimated using STEM-EELS maps corresponding to **a**. The element composition profile for oxygen provides further evidence for the oxygen ions diffusion from STO to SFO under the combined influence of elevated temperature and in situ TEM illumination.

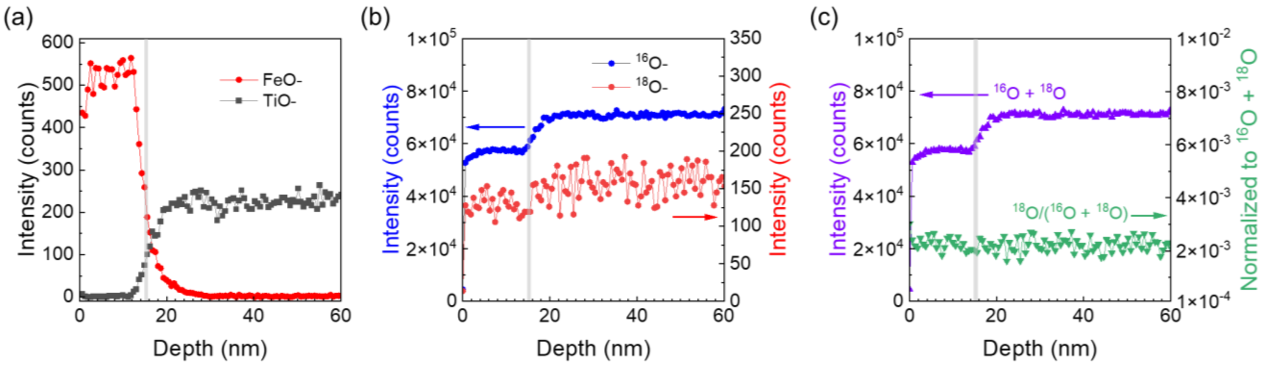

Supplementary Figure 5 | Examining the influence of potential matrix effects on interface oxygen intensity of a non-$^{18}O_2$ annealed BM-SFO(≈15 nm)/STO. **a**, The interface location of SFO/STO was confirmed by the secondary ion signals of FeO- and TiO-. **b**, Depth profiles of the ^{16}O- and ^{18}O- signals obtained via ToF-SIMS. **c**, Depth profiles of the total oxygen signal (purple) and normalized ^{18}O- signal (green). The vertical grey lines denote the interface.

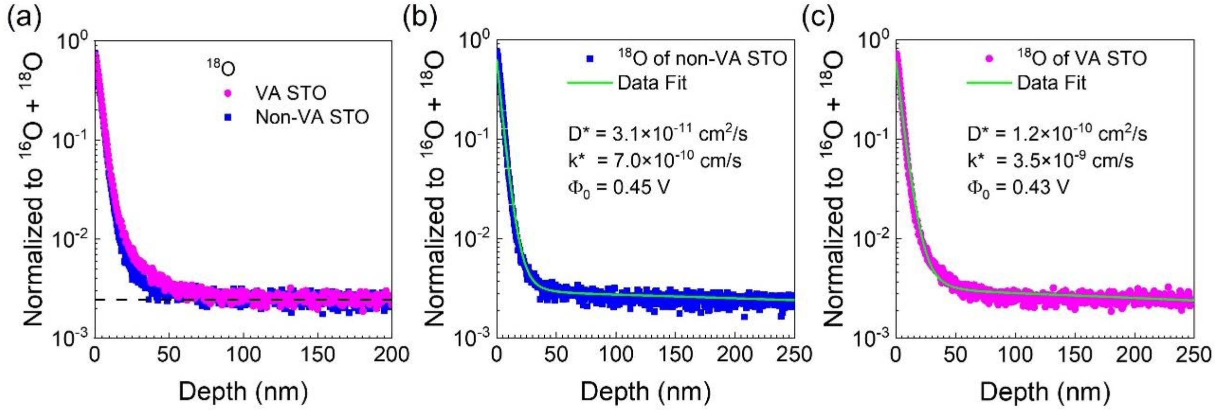

Supplementary Figure 6 | Determination of the bulk oxygen diffusion coefficient (D*) in STO. **a,** ToF-SIMS ^{18}O- depth profiles for VA STO and non-VA STO substrates. The black dashed line denotes the ^{18}O experimental natural abundance level. **b,c,** Data fitting for (b) non-VA and (c) VA STO substrates, where k^* is the surface exchange coefficient and Φ_0 is the space-charge potential.

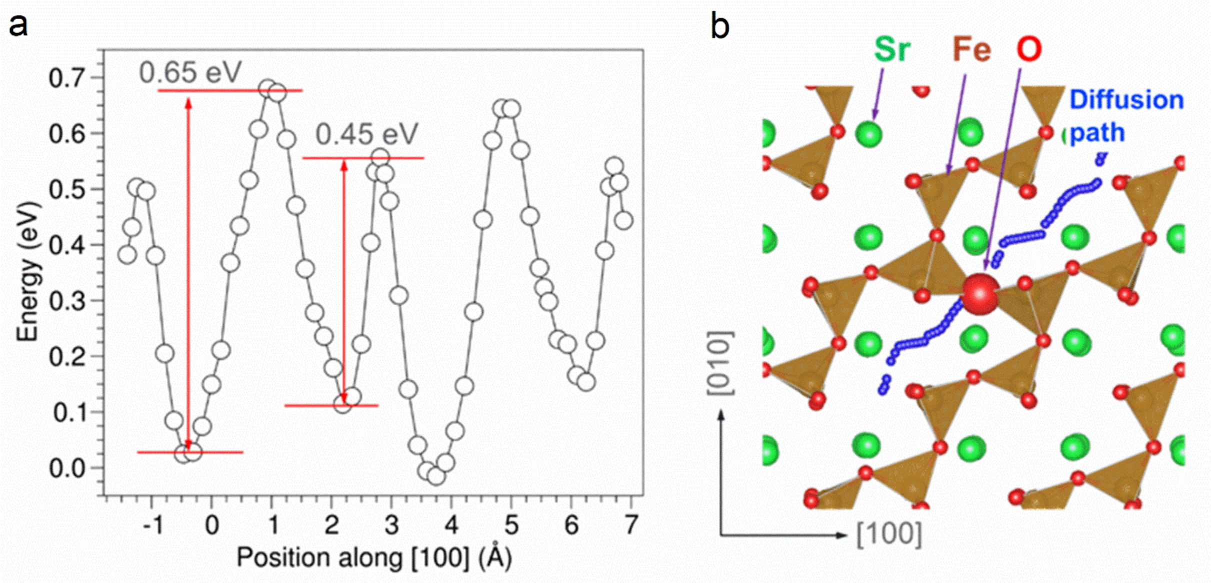

Supplementary Figure 7 | Diffusion of O^{2-} in the BM-SFO vacancy channels parallel to the substrate surface (the case of BM-SFO on STO). a, Calculated potential energy surface for O^{2-} diffusion in the strained BM-SFO. **b**, A fragment of the diffusion pathway. Only the FeO plane of the BM-SFO lattice and neighboring SrO planes are shown. Sr, Fe, and O atoms are shown with green, orange, and red spheres, respectively. The additional oxygen atom is shown with a large red sphere; small dark blue spheres indicate points along the diffusion path.

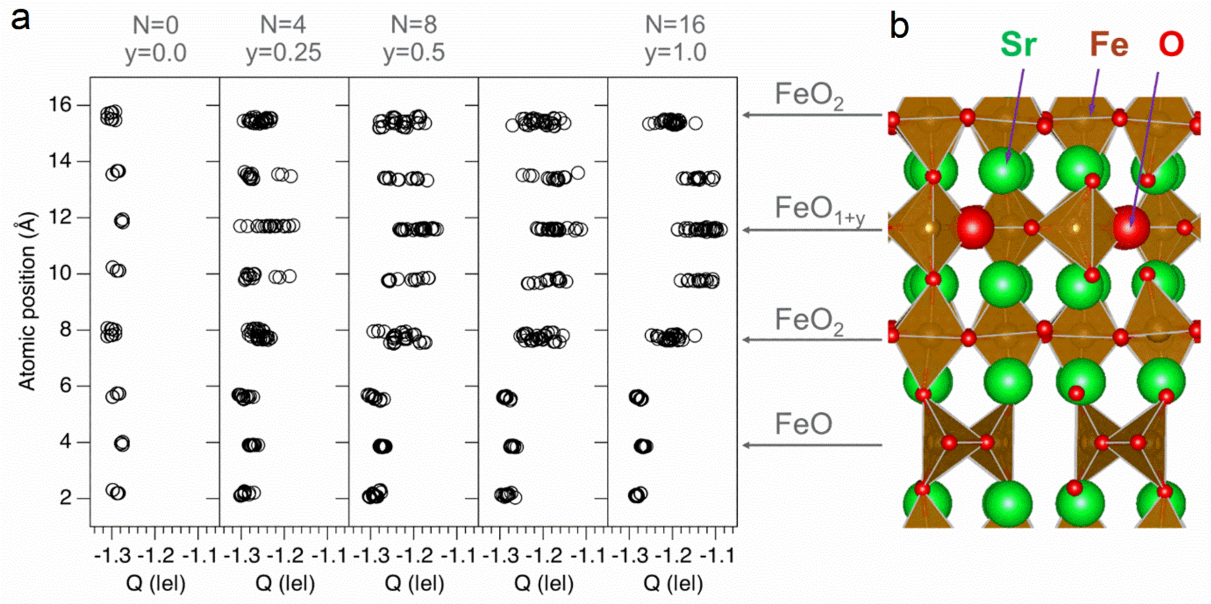

Supplementary Figure 8 | Modification of the charge density distribution upon oxidation of SFO_x. a, Effect of oxygen incorporation on the Bader charges Q of oxygen ions (|e| indicates the absolute value of the electron charge e) as a function of the number of oxygen atoms (N) incorporated into a single FeO_{1+y} ($0 \leq y \leq 1$) plane. **b**, Structural model of the SFO_x lattice with the OVCs oriented parallel to the substrate surface. Sr, Fe, and O atoms are shown with green, orange, and red spheres, respectively. Additional oxygens are shown as large red spheres. As N increases from 0 to 16, the local atomic structure evolves from BM-SFO (y=0) to P-SFO (y=1) and ions in the oxygen-rich region become less negative.

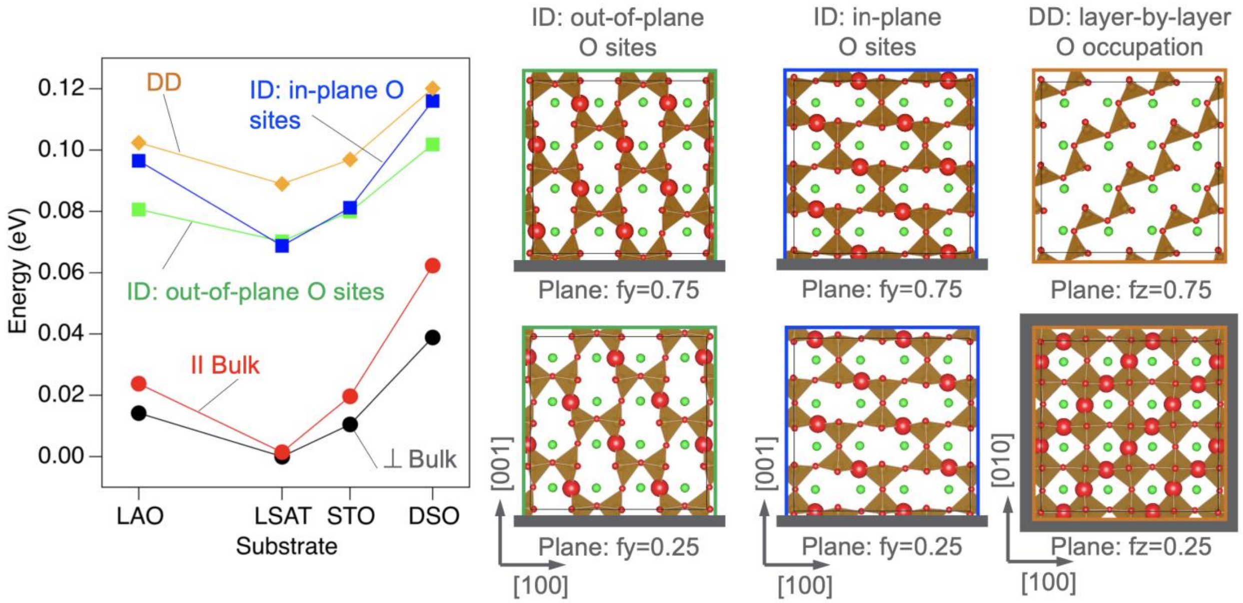

Supplementary Figure 9 | Stability and structure of the $SrFeO_{2.75}$ phases. Relative stability of the nominal $SrFeO_{2.75}$ phases (energy per formula unit) calculated as a function of substrate-induced strain (left) and the corresponding structural configurations of the OVC planes partially occupied with additional Oxygens shows with large spheres (right). In all cases, substrate strain was applied in the [100]-[010] plane; the location of the substrate is indicated with gray bars. In-plane lattice parameters of the $SrFeO_{2.75}$ phases were fixed at the values 3.790, 3.868, 3.905, and 3.950 Å to reproduce strain induced by the LAO, LSAT, STO, and DSO substrates, respectively. Bulk-like phases with oxygen vacancies rows oriented in out-of-plane and in-plane directions are the most stable among all considered $SrFeO_{2.75}$ phases. Intercalation diffusion (ID) phases have OVC planes oriented perpendicular to the substrate and fractional coordinates along the [010] direction fy=0.25 and fy=0.75; 50% of the vacant sites in each OVC is occupied with the additional oxygens. Two ID phases corresponds to the additional oxygens located at the out-of-plane (O-oop) and in-plane (O-ip) oxygen sites, respectively. Phase corresponding to disruptive diffusion (DD) has OVC planes stack along the [001] direction with fractional coordinates fz=0.25 and fz=0.75. In the considered $SrFeO_{2.75}$ phase, one OVC plane has 100% of the vacant sites occupied (fz=0.25) and the other OVC plane has no additional oxygens (fz=0.75).

Supplementary references

1 Wang, L., Yang, Z. Z., Bowden, M. E. & Du, Y. G. Brownmillerite phase formation and evolution in epitaxial strontium ferrite heterostructures. Appl. Phys. Lett. **114**, 231602 (2019).

2 Yao, L. et al. Electron-beam-induced Perovskite-Brownmillerite-Perovskite structural phase transitions in epitaxial $La_{2/3}Sr_{1/3}MnO_3$ films. Adv. Mater. **26**, 2789-2793 (2014).

3 Jang, J. H. et al. In Situ Observation of Oxygen Vacancy Dynamics and Ordering in the Epitaxial $LaCoO_3$ System. ACS Nano **11**, 6942-6949 (2017).

4 Das, T., Nicholas, J. D. & Qi, Y. Long-range charge transfer and oxygen vacancy interactions in strontium ferrite. J. Mater. Chem. A **5**, 4493-4506 (2017).
