# Supplementary material for: Guided anisotropic oxygen transport in vacancy ordered oxides
Source: Nat Commun. 2023 Sep 28;14:6068. doi: 10.1038/s41467-023-40746-4 (PMC10539514; doi:10.1038/s41467-023-40746-4)
Supplement: Supplementary file 3 — Description of Additional Supplementary Files [file 41467_2023_40746_MOESM3_ESM.pdf]

Description of Additional Supplementary Files

Supplementary Movie 1:

In-situ atomic-scale observation of dynamical phase transition processes in BM-SFO/STO under simultaneously applied 300 °C sample heating and electron beam illumination. The video is accelerated by the factor of 20 relative to the actual time.

Supplementary Movie 2:

In-situ atomic-scale observation of nucleation and phase transition processes in BM-SFO/STO under simultaneously applied 200 °C sample heating and electron beam illumination. The video is 15 times faster than actual time.

Supplementary Movie 3:

In-situ observation of phase transition processes in BM-SFO/LSAT under simultaneously applied 200 °C sample heating and electron beam illumination. The video is 5 times faster than actual time.

Supplementary Movie 4:

In-situ observation of phase transition processes in BM-SFO/LSAT under simultaneously applied 200 °C sample heating and electron beam illumination. The video is 5 times faster than actual time.
