# Supplementary material for: Guided anisotropic oxygen transport in vacancy ordered oxides
Source: Nat Commun. 2023 Sep 28;14:6068. doi: 10.1038/s41467-023-40746-4 (PMC10539514; doi:10.1038/s41467-023-40746-4)
Supplement: Supplementary file 8 — Reporting Summary [file 41467_2023_40746_MOESM8_ESM.pdf]

nature portfolio

Corresponding author(s): Peter V. Sushko; Yingge Du

Last updated by author(s): Jul 20, 2023

Reporting Summary

Nature Portfolio wishes to improve the reproducibility of the work that we publish. This form provides structure for consistency and transparency in reporting. For further information on Nature Portfolio policies, see our Editorial Policies and the Editorial Policy Checklist.

Statistics

For all statistical analyses, confirm that the following items are present in the figure legend, table legend, main text, or Methods section.

n/a	Confirmed	
☐	☒	The exact sample size (*n*) for each experimental group/condition, given as a discrete number and unit of measurement
☐	☒	A statement on whether measurements were taken from distinct samples or whether the same sample was measured repeatedly
☒	☐	The statistical test(s) used AND whether they are one- or two-sided<br>*Only common tests should be described solely by name; describe more complex techniques in the Methods section.*
☒	☐	A description of all covariates tested
☒	☐	A description of any assumptions or corrections, such as tests of normality and adjustment for multiple comparisons
☐	☒	A full description of the statistical parameters including central tendency (e.g. means) or other basic estimates (e.g. regression coefficient) AND variation (e.g. standard deviation) or associated estimates of uncertainty (e.g. confidence intervals)
☒	☐	For null hypothesis testing, the test statistic (e.g. *F*, *t*, *r*) with confidence intervals, effect sizes, degrees of freedom and *P* value noted<br>*Give P values as exact values whenever suitable.*
☒	☐	For Bayesian analysis, information on the choice of priors and Markov chain Monte Carlo settings
☒	☐	For hierarchical and complex designs, identification of the appropriate level for tests and full reporting of outcomes
☒	☐	Estimates of effect sizes (e.g. Cohen's *d*, Pearson's *r*), indicating how they were calculated

Our web collection on statistics for biologists contains articles on many of the points above.

Software and code

Policy information about availability of computer code

Data collection	DigitalMicrograph 3 software, Vienna Ab initio Software Package (VASP)
Data analysis	DigitalMicrograph 3 software

For manuscripts utilizing custom algorithms or software that are central to the research but not yet described in published literature, software must be made available to editors and reviewers. We strongly encourage code deposition in a community repository (e.g. GitHub). See the Nature Portfolio guidelines for submitting code & software for further information.

Data

Policy information about availability of data

All manuscripts must include a data availability statement. This statement should provide the following information, where applicable:

- Accession codes, unique identifiers, or web links for publicly available datasets
- A description of any restrictions on data availability
- For clinical datasets or third party data, please ensure that the statement adheres to our policy

The data supporting the findings of this study are available within the paper and its supplementary information files.

Research involving human participants, their data, or biological material

Policy information about studies with human participants or human data. See also policy information about sex, gender (identity/presentation), and sexual orientation and race, ethnicity and racism.

Reporting on sex and gender	N/A
Reporting on race, ethnicity, or other socially relevant groupings	N/A
Population characteristics	N/A
Recruitment	N/A
Ethics oversight	N/A

Note that full information on the approval of the study protocol must also be provided in the manuscript.

Field-specific reporting

Please select the one below that is the best fit for your research. If you are not sure, read the appropriate sections before making your selection.

☐ Life sciences ☐ Behavioural & social sciences ☒ Ecological, evolutionary & environmental sciences

For a reference copy of the document with all sections, see nature.com/documents/nr-reporting-summary-flat.pdf

Ecological, evolutionary & environmental sciences study design

All studies must disclose on these points even when the disclosure is negative.

Study description	In this study, by using in situ transmission electron microscopy, we directly show the oxygen ion migration dynamics in vacancy ordered semiconducting SrFeO2.5 epitaxial thin films at atomic scale.
Research sample	Strontium ferrite (SrFeOx) is a convenient platform to study reversible redox activity and associated property changes. Oxygen-deficient brownmillerite-structured SrFeO2.5 (BM-SFO), with ordered oxygen vacancy channels, can exhibit facile, highly anisotropic oxygen ion transport and low energy diffusion barriers within the OVCs, making these materials attractive for use as electrocatalysts and oxygen membranes.
Sampling strategy	Epitaxial SrFeO2.5 thin films with the thickness of 15-30 nm were grown on (001)-oriented SrTiO3 and LSAT single crystal substrates by using PLD. The TEM samples used for in situ TEM were prepared using a focus ion beam scanning electron microscopy (Helios).
Data collection	Z. Yang collects in situ TEM data in Titan TEM using a Gatan heating holder. The movie is acquired during the in situ TEM experiment, and the fine structural analysis is performed before and after the in situ experiment using atomic resolution HAADF imaging.
Timing and spatial scale	The TEM data is collected between November 2018 and June 2020. The computation data is gathered between June 2020 and May 2022. SIMS data is collected from June 2019 to June 2020, then from December 2022 to April 2023.
Data exclusions	No data were excluded from the analyses.
Reproducibility	The results of the experiment are repeatable.
Randomization	The sample we used are epitaxial BM-SFO thin films deposited on (001)-oriented SrTiO3 and (LaAlO3)0.3(Sr2AlTaO6)0.7 single crystal substrates by pulsed laser deposition (PLD). Therefore, the thin film samples are grouped by substrates.
Blinding	During the in situ TEM experiment, the video was taken in real time. Therefore, the blinding is not relevant to our study.

Did the study involve field work? ☐ Yes ☒ No

Reporting for specific materials, systems and methods

We require information from authors about some types of materials, experimental systems and methods used in many studies. Here, indicate whether each material, system or method listed is relevant to your study. If you are not sure if a list item applies to your research, read the appropriate section before selecting a response.

Materials & experimental systems

n/a	Involved in the study
☒	☐ Antibodies
☒	☐ Eukaryotic cell lines
☒	☐ Palaeontology and archaeology
☒	☐ Animals and other organisms
☒	☐ Clinical data
☒	☐ Dual use research of concern
☒	☐ Plants

Methods

n/a	Involved in the study
☒	☐ ChIP-seq
☒	☐ Flow cytometry
☒	☐ MRI-based neuroimaging
